# Combining Hypoxia and Bioreactor Hydrodynamics Boosts Induced Pluripotent Stem Cell Differentiation Towards Cardiomyocytes

**DOI:** 10.1007/s12015-014-9533-0

**Published:** 2014-07-15

**Authors:** Cláudia Correia, Margarida Serra, Nuno Espinha, Marcos Sousa, Catarina Brito, Karsten Burkert, Yunjie Zheng, Jürgen Hescheler, Manuel J. T. Carrondo, Tomo Šarić, Paula M. Alves

**Affiliations:** 1 Instituto de Tecnologia Química e Biológica, Universidade Nova de Lisboa, Av. da República, Oeiras, 2780-157 Portugal; 2 iBET, Instituto de Biologia Experimental e Tecnológica, Apartado 12, 2780-901 Oeiras, Portugal; 3 Institute for Neurophysiology, Medical Faculty, University of Cologne, Cologne, Germany; 4 Faculdade de Ciências e Tecnologia, Universidade Nova de Lisboa, 2829-516 Caparica, Portugal; 5 Center for Physiology and Pathophysiology, Institute for Neurophysiology, University of Cologne, Robert Koch Str. 39, 50931 Cologne, Germany; 6 Animal Cell Technology Unit, Instituto de Tecnologia Química e Biológica, Universidade Nova de Lisboa and iBET, Instituto de Biologia Experimental e Tecnológica, Apartado 12, 2781-901 Oeiras, Portugal

**Keywords:** Induced pluripotent stem cells, Cardiomyocyte differentiation, Hypoxia, Mechanical environment, Bioreactor hydrodynamics, Mass production

## Abstract

Cardiomyocytes (CMs) derived from induced pluripotent stem cells (iPSCs) hold great promise for patient-specific disease modeling, drug screening and cell therapy. However, existing protocols for CM differentiation of iPSCs besides being highly dependent on the application of expensive growth factors show low reproducibility and scalability. The aim of this work was to develop a robust and scalable strategy for mass production of iPSC-derived CMs by designing a bioreactor protocol that ensures a hypoxic and mechanical environment. Murine iPSCs were cultivated as aggregates in either stirred tank or WAVE bioreactors. The effect of dissolved oxygen and mechanical forces, promoted by different hydrodynamic environments, on CM differentiation was evaluated. Combining a hypoxia culture (4 % O_2_ tension) with an intermittent agitation profile in stirred tank bioreactors resulted in an improvement of about 1000-fold in CM yields when compared to normoxic (20 % O_2_ tension) and continuously agitated cultures. Additionally, we showed for the first time that wave-induced agitation enables the differentiation of iPSCs towards CMs at faster kinetics and with higher yields (60 CMs/input iPSC). In an 11-day differentiation protocol, clinically relevant numbers of CMs (2.3 × 10^9^ CMs/1 L) were produced, and CMs exhibited typical cardiac sarcomeric structures, calcium transients, electrophysiological profiles and drug responsiveness. This work describes significant advances towards scalable cardiomyocyte differentiation of murine iPSC, paving the way for the implementation of this strategy for mass production of their human counterparts and their use for cardiac repair and cardiovascular research.

**Electronic supplementary material:**

The online version of this article (doi:10.1007/s12015-014-9533-0) contains supplementary material, which is available to authorized users.

## Introduction

The inability of mature cardiomyocytes (CMs) to proliferate leads to a permanent loss of functional cells after injury [1]. Previous studies in animal models of myocardial infarction have demonstrated that the function of a damaged heart may be improved by transplantation of sufficient numbers of functional CMs [2]. Over the last years, pluripotent stem cells (PSCs), including embryonic stem cells (ESCs) and induced pluripotent stem cells (iPSCs), have emerged as an attractive candidate stem cell source for obtaining CMs [3, 4]. The inherent capacity to grow indefinitely and to differentiate into all mature cells of the human body make PSCs the only cell source that can provide ex-vivo an unlimited number of functional and potentially autologous CMs for transplantation. The clinical translation of human ESC-derivatives has been greatly hampered by the risk of immune rejection due to their allogenicity and by ethical concerns [5]. iPSCs can circumvent these drawbacks, allowing for ethically “acceptable” and safe patient-specific therapies [6, 7]. Moreover, iPSCs constitute a promising tool to establish disease-specific models of human inherited cardiac disorders and platforms for drug discovery and toxicity testing [7, 8].

In the last 5 years, several methodologies have been described for the differentiation of murine [9–12] and human [13–18] iPSCs into functional CMs based on the knowledge acquired in previous studies with ESCs. Still, several challenges remain that currently preclude their widespread application. Those protocols typically involve a complex stage-specific application of exogenous growth factors which are costly, degrade rapidly, do not readily diffuse into complex 3D aggregates and exhibit lot-to-lot variation in their bioactivity [19]. Moreover, despite recent improvements in cardiac differentiation protocols [15–18] these are still associated with low reproducibility and scalability [5], being unsuitable to provide the large numbers of CMs needed to exert functional benefit after a heart attack (about 1–2 × 10^9^ CMs per patient) [20]. Therefore, robust and scalable bioprocesses for CM production less dependent on the use of inductive factors are required for a faster transition of iPSCs to the clinical and industrial fields.

One of the most powerful strategies for scaling-up the production of iPSC derivatives consists in cultivating the cells as 3D cell aggregates called embryoid bodies in bioreactor systems that continuously assure monitoring and control of the environmental conditions (pH, pO_2_ and agitation profile) [21, 22]. A close control of the physical environment was shown to be essential for guiding cell fate decisions through expansion and differentiation routes. Low oxygen tensions (2–5 % O_2_) have been shown to enhance the proliferation of PSCs [22–24] and their differentiation to CMs [25–27]. Indeed, it is well known that cells in the early developing embryo are exposed to low oxygen levels. In hamsters and rabbits, for example, intrauterine oxygen concentrations decrease during blastulation and implantation to 5.3 % O_2_ and 3.5 % O_2_, respectively [28]. Thus, lowering the oxygen concentration from normoxic atmospheric levels (20 % O_2_) to more physiological levels (2–5 % O_2_ or atmospheric hypoxia) might be beneficial in PSC cultures due to the importance of this environmental condition during embryonic development.

Mechanical cues from the environment are also translated into biological signals that mediate cell structure, survival, migration, proliferation, and differentiation [29, 30]. Several studies have provided evidence that applied mechanical forces including cyclic strain and stretch, fluid shear stress and hydrostatic compression modulate differentiation of cells that reside in mechanically dynamic environments, such as CMs [31–33], vascular smooth muscle cells [34], endothelial cells [35] and chondrocytes [36]. In particular, CMs are continuously subjected to cyclic mechanical strains promoted by the rhythmic heart beating [37]. CM-enriched tissue constructs are often subjected to cyclic tensions to increase their force of contraction, facilitate organization of cellular structures and consequently improve their cardiac function in vivo [38]. Thus, the hypothesis that mechanical loading promotes cardiomyogenesis of PSCs has started to be explored [31, 39, 40]. It was recently reported that ESCs cultured on elastic polymer [poly (lactide-co-caprolactone), PLCL] scaffolds and subjected to 1 % cyclic uniaxial stretch, in a custom-made strain device, demonstrated commitment towards CM lineage as noted by an increased cardiac gene expression compared to unstrained controls [31]. Nevertheless, the low high-throughput design and scalability of these devices, custom built to apply mechanical strains, makes them unsuitable to generate large numbers of cells on a therapeutically relevant scale.

In the present work we focused on the development of robust, scalable and integrated platforms for iPSC-derived CM production and purification using bioreactor systems with automated process control, including online measurement and adjustment of the culture parameters. Our strategy consisted on modulating key environmental parameters for efficient and reliable differentiation of iPSC towards the CM lineage, reducing the need of using inductive factors. We explored the impact of dissolved oxygen (DO) and mechanical forces on CM differentiation of iPSCs by using two distinct bioreactor systems, namely stirred tank and WAVE bioreactors. We applied mechanical forces to cells by manipulating the hydrodynamic environment, more specifically the type and profile of agitation. We describe for the first time a protocol for efficient mass production of functional CMs derived from murine iPSCs that combines a hypoxic environment with an intermittent stirring or a wave-induced agitation profile.

## Methods

### iPSC Culture on Feeder Layers

A murine transgenic αPIG-iPS cell line, in which the puromycin-N-acetyl transferase and the enhanced green fluorescent protein (eGFP) genes are under the control of the cardiac-specific alpha-myosin heavy chain (α-MHC) promoter [41], was used in this study to facilitate bioprocess development. The fluorescence marker and the antibiotic resistance genes, specifically expressed in CMs, allow easy monitoring of the cardiac differentiation process and selection of a highly pure CM population upon addition of puromycin into the media, respectively. iPSCs were cultivated on a monolayer of mitotically inactivated murine embryonic fibroblasts (MEFs) in Dulbecco’s modified Eagle medium (DMEM) supplemented with 15 % (v/v) fetal bovine serum (FBS), 1 % (v/v) non-essential amino acids (NEAA), 2 mM L-glutamine, 50 μM β-mercaptoethanol, 500 μg/mL neomycin sulfate (all from Invitrogen, UK), and 1000 U/mL leukemia inhibitory factor (LIF) (ESGRO, Merck Millipore, Germany), at 37 ºC in a humidified atmosphere of 5 % CO_2_. Cells were passaged every two days as previously reported [41].

### iPSC Differentiation in Stirred Tank Bioreactors

**Fig. 1:**
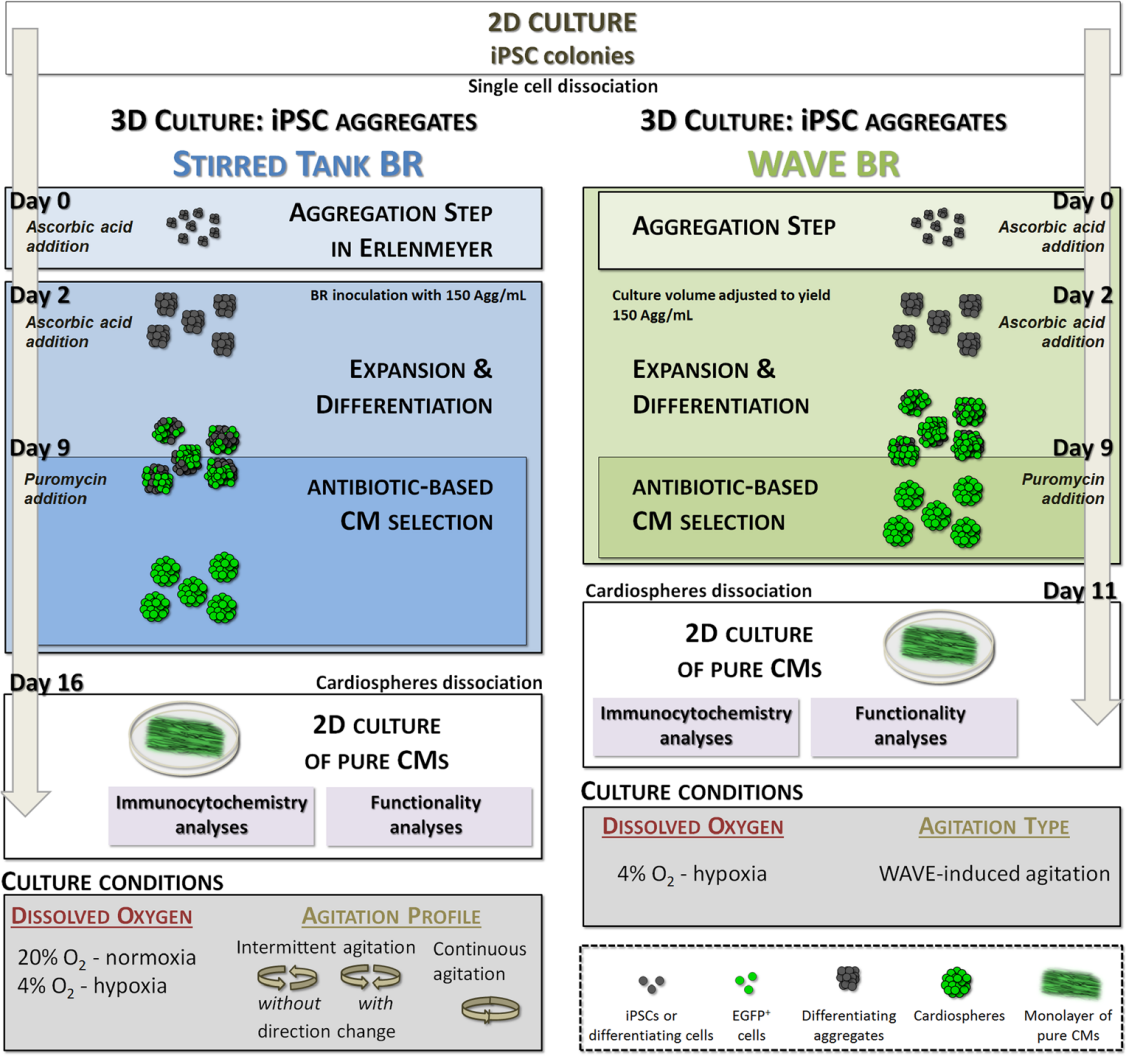
Experimental scheme for differentiation and purification of iPSC-derived CMs in stirred tank and WAVE bioreactors. In stirred tank bioreactors, an aggregation step was first performed in an Erlenmeyer for 48 h. After this time, aggregates were transferred to bioreactors to yield 150 aggregates/mL and cultured in the presence of ascorbic acid for additional 7 days. At day 9, puromycin was added to the medium to eliminate non-CMs. After 7 days of antibiotic-based CM selection, pure CM aggregates (cardiospheres) were harvested, dissociated and CMs were cultured in 2D plates for characterization studies (immunofluorescence microscopy and electrophysiological studies). In WAVE bioreactor cultures, cells were inoculated as single cells directly into the WAVE bioreactor and at day 2 the culture volume was adjusted to obtain 150 aggregates/mL. Aggregates were cultured in the presence of ascorbic acid for additional 7 days. At day 9, CM lineage selection was initiated and lasted 2 days. At day 11, aggregates were harvested, dissociated and plated in 2D plates for further characterization. Culture conditions evaluated in each bioreactor system are depicted in the bottom of the schematic workflow

To promote cell aggregation 0.7 × 10^5^ cell/mL were inoculated into plastic Erlenmeyer flasks (Corning, USA) containing 100 mL of differentiation medium (Iscove’s modified Dulbecco’s medium (IMDM) with GlutaMAX, supplemented with 20 % (v/v) FBS, 1 × NEAA, 1 % (v/v) Pen/Strep, 50 μM β-mercaptoethanol (all from Invitrogen, UK) and 100 μM ascorbic acid (Wako, Germany), and incubated at 37 ºC in a 5 % CO_2_ humidified atmosphere on an orbital shaker at 80–90 rpm. After two days aggregates were transferred into stirred tank bioreactors (DasGip cellferm-pro bioreactor system, Germany) and cultured at a concentration of 150 aggregates/mL in 200 mL of differentiation medium. Medium was partially changed at days 9 (50 % v/v), 12 (70 % v/v) and 14 (50 % v/v) by selection medium (differentiation media without ascorbic acid supplemented with puromycin at a final concentration of 8 μg/mL (InvivoGen, USA)) to eliminate non-CMs and promote CM selection. Antibiotic treatment resulted in the generation of pure aggregates of CMs (designated hereafter as cardiospheres). The experimental set up is illustrated in Fig. 1. All cultures were performed in computer-controlled stirred tank bioreactors equipped with a trapezoid shaped paddle impeller with arms and operated under defined conditions (CO_2_: 5 %; temperature: 37 °C; DO: 20 % O_2_ tension (atmospheric normoxia) or 4 % O_2_ tension (atmospheric hypoxia); surface aeration rate: 0.1 vvm (gas volume flow per unit of liquid volume per minute); agitation rate: 90 rpm for complete continuous stirred-tank reactor (CSTR) behavior; agitation profile: continuous or intermittent (ON: 30 s, OFF: 0 s) with or without direction change; cyclic mechanical frequency (defined by the number of stirring interruptions per unit of time): 0.033Hz). Data acquisition and process control were performed using DasGip Control Software 4.0. Three independent bioreactor runs were performed for every experimental setting.

### iPSC Differentiation in WAVE Bioreactors

Cell aggregation was promoted in Cellbag^TM^—WAVE bioreactors (GE Healthcare, Sweden) for 2 days (Fig. 1). Briefly, 0.7 × 10^5^ cell/mL were inoculated as single cells into WAVE bioreactors containing 500 mL of differentiation medium. At day 2, aggregate concentration was adjusted to 150 aggregates/mL by increasing the working volume to 1 L. At day 9 of differentiation, CM purification was initiated by exchanging half of the culture media with selection medium. Aggregates were cultivated for additional 2 days in these conditions. The experimental set up is shown in Fig. 1. All cultures were performed in computer-controlled WAVE bioreactor systems under defined conditions (CO_2_: 5 %; temperature: 37 °C; DO: 4 % O_2_ tension; surface aeration rate: 0.1 vvm; rocking angle: 4; rocking rate (adjusted throughout culture time according to the aggregate size): 10 rocks/min (day0–day1), 12 rocks/min (day1–day2), 25 rocks/min (day2–day7), 26 rocks/min (day7–day11); cyclic mechanical frequency from day 2 to day 11 (defined by the change in wave motion per unit of time): 0.82 to 0.86 Hz). Data acquisition and process control were performed using UNICORN* DAQ 1.0 software. Three independent bioreactor runs were performed for every experimental setting.

### Dissociation of Cardiospheres

At the end of the differentiation process, cardiospheres were harvested from the bioreactor, dissociated to single cells by incubation with 0.25 % (w/v) Trypsin-EDTA (Invitrogen, UK) for 5–7 min at 37 °C and transferred to CELLstart™ (Invitrogen, UK) coated 6- or 24-well plates for further characterization.

Methods for evaluation of cell and aggregate concentration, monitorization of CM differentiation and characterization of iPSC-derived CMs are provided in the supplementary data supplement.

## Results

### Effect of Dissolved Oxygen on CM Differentiation of iPSCs in Stirred Tank Bioreactors

In order to establish a robust and scalable platform for production of functional iPSC-derived CMs, we first investigated the effect of DO on cardiac iPSC differentiation in stirred tank bioreactor systems. For that purpose, we compared the effect of normoxic atmospheric oxygen levels (20 % O_2_ tension) with physiological oxygen levels (2–5 % O_2_ tension or atmospheric hypoxia). Within this range of physiological oxygen levels, we selected a value of 4 % O_2_ since previously reported studies showed that this oxygen concentration improves CM differentiation in ESC aggregate cultures [25, 26].

Aggregation of single cell suspension was promoted in Erlenmeyers and orbital agitation for 48 h under normoxia conditions; these conditions resulted in the formation of small aggregates with a mean size of 155 ± 56 μm that readily metabolized the vital dye fluorescein diacetate but not the dead cell marker propidium iodide (Supplementary Fig. I). These aggregates were transferred to fully controlled stirred tank bioreactors and cultured under either normoxic (DO = 20 % O_2_) or hypoxic (DO = 4 % O_2_) conditions for additional 14 days (Fig. 1).

**Fig. 2:**
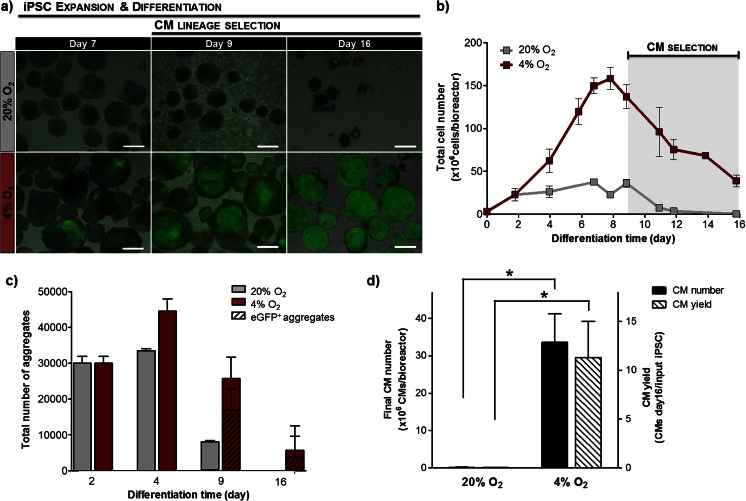
Effect of DO on CM differentiation of iPSCs in stirred tank bioreactors. Aggregates were inoculated at day 2 in stirred tank bioreactors at DO of 20 % O_2_ (atmospheric normoxia) or 4 % O_2_ (atmospheric hypoxia). **a**. Merged phase contrast and fluorescence images showing cell aggregates composed by eGFP-positive cells (*green*) at days 7, 9 and 16 of culture. *Scale bars*: 200 μm. **b**. Profile of total cell number during culture time. **c**. Total number of aggregates at days 2, 4, 9 and 16. The number of eGFP-positive aggregates is indicated by *striped bars*. **d**. Total number of CMs produced per bioreactor run (*black bars*) and CM yield (number of CMs at day 16/input iPSC, *striped bars*) obtained in both normoxic and hypoxic culture conditions. *Error bars* (**b**–**d**) represent SD of 3 individual bioreactor experiments. Significantly different, *P* < 0.05 (*) and *P* < 0.01 (**)

The results showed that a DO of 4 % O_2_ maximizes cell proliferation and CM differentiation (Fig. 2). In these culture conditions, the size of aggregates, the total number of cells and aggregates were higher throughout the culture (Fig. 2a–c) resulting in a significantly higher expansion fold on day 9 of differentiation when compared to normoxic conditions (44.9 ± 2.7 versus 12.2 ± 1.4 cells/initial iPSC, *p* = 0.001, Table 1). Moreover, first eGFP-positive cells and contracting areas in aggregates cultured under hypoxia were observed already on day 7 of differentiation, contrasting with cell aggregates cultured at 20 % O_2_ where eGFP-positive cells and beating areas were rarely observed throughout culture time (Fig. 2a). The higher overall cell expansion in hypoxic cultures corresponded to a significant improvement in CM yield as determined on day 16 of differentiation, after 7 days of puromycin selection (11.3 ± 3.7 vs. 0.05 ± 0.02 CMs/input iPSC, *p* = 0.045, Fig. 2d), and to an approximately 210-fold enhancement in CM numbers compared to normoxia (33.6 ± 7.6 × 10^6^ CMs vs. 0.15 ± 0.06 × 10^6^ CMs per bioreactor run, *p* = 0.01, Fig. 2d). Since a low DO environment proved to be a key parameter in enhancing CM differentiation of iPSCs, we selected this culture condition to be used in further experiments.

**Table 1:** Quantitative characterization of CM differentiation of iPSC in automated stirred tank and WAVE bioreactors

Culture system	Stirred tank bioreactor			WAVE bioreactor
Agitation type and profile	Continuous	Intermittent with direction change	Intermittent without direction change	Wave-induced
Working volume (mL) Cyclic strain frequency (Hz)	200 0	200 0.033	200 0.033	1000 0.82 to 0.86
Expansion fold (cells on day 9/input iPSC)	44.9 ± 2.7	23.7 ± 1.7	73.4 ± 7.6	65.7 ± 7.2
CM purity before purification (day 9, %eGFP-positive cells)	23.3 ± 5.0	16.5 ± 3.7	43.9 ± 6.6	76.0 ± 5.8
CM purity after selection (day 16/11, %eGFP-positive cells)	85.7 ± 4.1	77.0 ± 3.0	97.4 ± 0.4	97.6 ± 1.0
Final CM number (×10^6^ CMs)	33.6 ± 7.6	19.5 ± 1.7	128.1 ± 3.3	2279.8 ± 69.2
Final CM yield (number of CMs/input iPSC)	11.3 ± 3.7	6.8 ± 1.1	44.0 ± 2.1	60.8 ± 0.7
Final CM number *per* liter of culture medium throughput (×10^6^ CMs/L)	78.1 ± 11.8	45.4 ± 4.0	295.9 ± 8.8	1561.1 ± 87.3

### Impact of Agitation Profile on CM Differentiation of iPSC in Stirred Tank Bioreactors

The effect of different agitation profiles on CM differentiation was assessed using stirred tank bioreactors operating under hypoxic conditions. A continuous agitation was compared with an intermittent agitation with and without change in the agitation direction. Based on previous reports that mechanical stimulation enhances contractile function and up-regulates cardiac gene expression [34, 39], we hypothesized that the hydrodynamic environment imposed by an intermittent agitation composed of repeated and brief stops could provide cyclic mechanical forces to the cells and consequently potentiate iPSC differentiation towards contractile CMs.

**Fig. 3:**
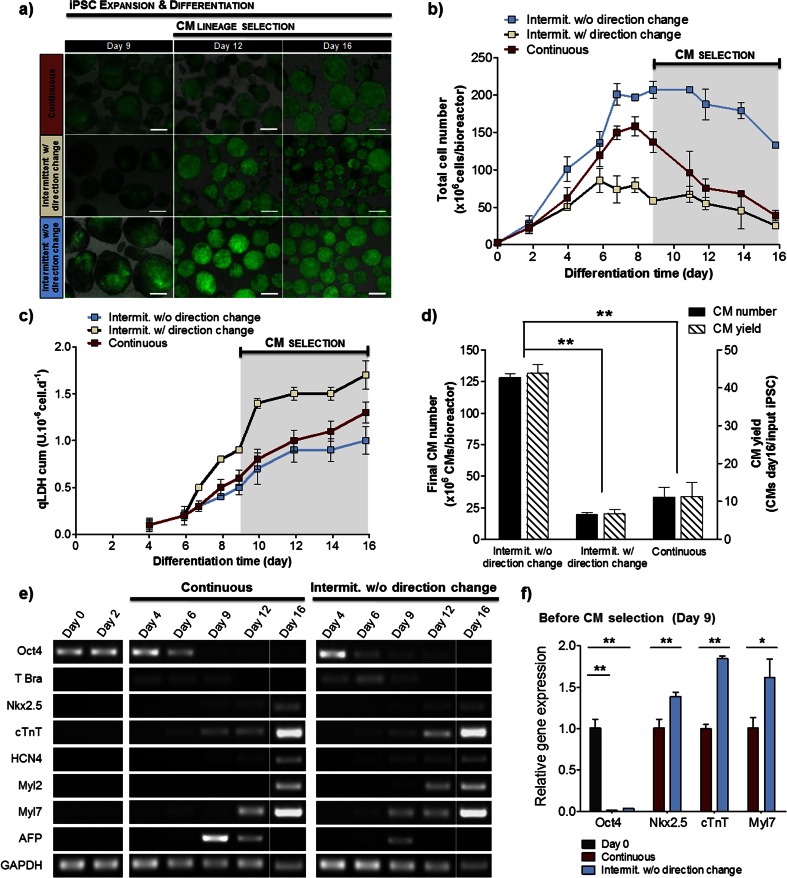
Impact of the agitation profile on CM differentiation of iPSCs in stirred tank bioreactors. Aggregates were inoculated at day 2 in stirred tank bioreactors operating in continuous and intermittent stirring with or without change of direction. **a**. Phase contrast and fluorescence images showing cell aggregates with eGFP-positive cells (*green*) at days 9, 12 and 16 of culture. Scale bars: 200 μm. **b**–**c**. Profile of total cell number (**b**) and cumulative values of specific rates of LDH release (**c**) in each bioreactor condition during culture time. **d**. Total number of CMs produced in each bioreactor run (*black bars*) and CM yields (*striped bars*). **e**. Semiquantitative RT-PCR analyses demonstrating the expression of pluripotency (Oct4), endoderm (AFP), early mesoderm (T-Brachyury) and CM-specific (Nkx2.5, cTNT, HCN4, Myl2, Myl7) genes in differentiating cells cultured in bioreactors operating in continuous agitation (*left panel*) and intermittent agitation without change of direction (*right panel*) profiles at different time points. **f**. Quantitative RT-PCR analysis showing the relative expression of Oct4, Nkx2.5, cTnT and Myl7 at day 9. Values were normalized to the continuous agitation culture condition, except Oct4 expression that was normalized to RNA from undifferentiated cells (day 0, *black bar*). Data are shown as mean ± SD of 3 individual bioreactor experiments. Significantly different, *P* < 0.05 (*) and *P* < 0.01 (**)

Our results show that an intermittent agitation profile without direction change led to a faster cell growth, higher cell number and enhanced CM differentiation when compared to the other agitation profiles evaluated (Fig. 3, Table 1). At day 9, an increase of 73.4 ± 7.6 fold in cell number was obtained in this culture condition (Table 1), reflecting more pronounced cell proliferation as compared to other two tested conditions. At this culture time point the percentage of eGFP-positive cells, as determined by flow cytometry analysis of dissociated aggregates (Supplementary Methods), was significantly higher in this culture condition (43.9 ± 6.6 %) than in continuous (23.3 ± 5.0 %, *p* = 0.01) or in intermittent agitation profile with direction change (16.5 ± 3.7 %, *p* = 0.01) cultures (Table 1), suggesting enhanced cardiac differentiation efficiency. In accordance, lower cell death during antibiotic treatment, as indicated by a lower accumulation of intracellular LDH in culture supernatant, was observed (Fig. 3c), indicating that a higher percentage of puromycin resistant iPSC-derived CMs and a lower amount of contaminating cells were present in culture. At the end of the process higher CM number (128.1 ± 3.3 × 10^6^ CMs/bioreactor), yield (44.0 ± 2.1 CMs/input iPSC) and purity (97.4 ± 0.4 %) were obtained in cultures operated under intermittent agitation without direction change (Fig. 3d, Table 1); in comparison to continuous agitation and intermittent agitation with direction change profiles, this culture condition enabled a significant improvement of 4- and 6.5-fold in CM production, respectively (*p* = 0.0001, Table 1).

RT-PCR analyses of RNA isolated from intermittent agitation profile without direction change and continuous agitation cultures reflect the impact of cyclic mechanical forces promoted by the different hydrodynamic environments on cell phenotype (Fig. 3e–f). A typical cardiac lineage gene expression pattern was observed in both culture conditions. The expression of the pluripotency marker Oct4 progressively decreased over the course of differentiation reaching very low levels by day 9 (Fig. 3e–f) and being undetectable at day 16 purified CMs (Fig. 3e). Additionally, the expression of the early mesoderm marker T brachyury was up-regulated from day 4 to day 6 and down-regulated thereafter. These results allow us to assume that the marked proliferation observed up to day 7 (Fig. 3b) in these cultures might have resulted from the prevalence of highly proliferative undifferentiated and mesodermal cells up to this timepoint. Afterwards, a progressive increase in the expression of cardiac-progenitor and cardiac-specific gene markers (Nkx2.5, cTnT, HCN4, Myl2 and Myl7) was observed. The higher band intensities at day 9 of differentiation (before initiation of CM selection) indicated that the majority of the cardiac-specific markers were greater expressed in cells cultured under intermittent agitation conditions. RT-qPCR analyses further confirmed that the expression of the cardiac genes Nkx2.5, cTnT and Myl7 in cells at this time point was, respectively, 1.4- (*p* < 0.01), 1.8- (*p* < 0.01) and 1.6-fold (*p* < 0.05) higher in cultures with intermittent agitation than in those with continuous agitation profile (Fig. 3f). In order to further assess the purity of puromycin-selected CM preparations, we monitored the expression of the endodermal marker α-fetoprotein (AFP) in cells from both cultures. AFP transcripts were detected on days 6–12 in cell aggregates from both conditions but their expression was higher in cultures with continuous agitation profile. On day 12, the expression of AFP was still observed in these cultures but not in cultures operated under intermittent agitation profile without direction change, thus suggesting that this agitation profile may provide a favorable hydrodynamic environment for cardiac but not endodermal lineage differentiation. Nonetheless, in both culture conditions the expression of AFP was not detectable at the end of the selection process (day 16), confirming the final purity of antibiotic selected CMs (Fig. 3e). Altogether, these results indicate that the mechanical stimulus provided to the cells by the agitation profile inherent to stirred-tank bioreactors can be modulated to enhance cardiomyogenesis.

### CM Differentiation of iPSCs in WAVE Bioreactors

In order to further explore the beneficial impact of mechanical forces induced by bioreactor hydrodynamics on CM differentiation, we evaluated the capacity of iPSCs to differentiate towards CMs in WAVE bioreactors.

Cell aggregation was initiated by inoculating single iPSCs into WAVE bioreactors. After 2 days of cultivation under hypoxic conditions aggregates presented a size of 167 ± 43 μm, similar to those obtained in Erlenmeyer flasks (Supplementary Fig. I).

**Fig. 4:**
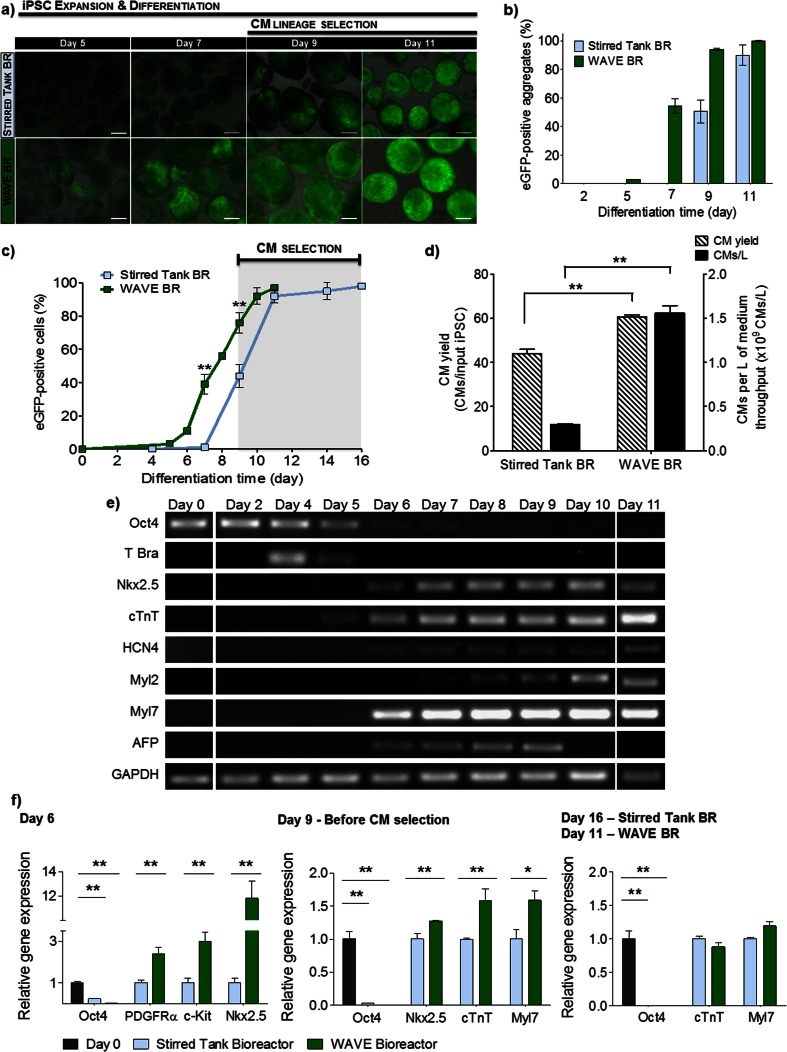
Differentiation of iPSC into CMs in WAVE and stirred tank bioreactors. WAVE cultures were compared with optimized stirred tank bioreactor cultures (operated under 4 % O_2_ and intermittent agitation without direction change) in terms of CM differentiation efficiency. **a**. Phase contrast and fluorescence images showing cell aggregates composed by eGFP-positive cells (*green*) at days 5, 7, 9 and 11. *Scale bars*: 200 μm. **c**. Percentage of eGFP-positive aggregates during culture time. **c**. Percentage of eGFP-positive cells during culture time determined by flow cytometry. **d**. CM yields (stripped bars) and number of CMs generated per liter of culture medium throughput (CMs/L, *black bars*). **e**. Semiquantitative RT-PCR analyses showing expression of pluripotency (Oct4), endoderm (AFP), mesoderm (T-Bra) and CM-specific (Nkx2.5, cTnT, HCN4, Myl2, Myl7) genes during the time course of differentiation process in WAVE bioreactors. **f**. Quantitative RT-PCR analysis of cells cultured in optimized stirred tank and WAVE bioreactors, at day 6, before (day 9) and after CM selection (day 16 and 11 for stirred tank and WAVE bioreactor cultures, respectively). Expression was normalized to RNA from stirred tank bioreactor cultures operating with intermittent agitation, except for Oct4 expression that was normalized to RNA of undifferentiated cells (day 0, *black bars*). Data are given as mean ± SD of 3 individual bioreactors experiments. Significantly different, *P* < 0.05 (*) and *P* < 0.01 (**)

The comparison of CM differentiation efficiency in WAVE and optimized stirred tank bioreactors (operated under 4 % O_2_ tension and intermittent agitation without direction change) revealed that WAVE bioreactor cultures favored CM lineage commitment, enabling a reduction in the differentiation time and increased CM yields (Fig. 4). In these cultures, eGFP-positive cells and contracting areas in aggregates were observed from day 5 onwards, increasing rapidly in number and size (Fig. 4a–c, Supplementary Movie I). By day 7, 54.3 ± 5.2 % of the aggregates exhibited a considerable area of eGFP-positive and spontaneously beating cells (Fig. 4a–b). In accordance with this result, flow cytometry analyses indicated that 38.6 ± 6.0 % of the cells in aggregates were eGFP-positive, compared to less than 5 % detected in stirred tank bioreactors at this time point (Fig. 4c). Importantly, on day 9 of culture, i.e. before CM selection, the percentage of eGFP-positive cells among all cells dissociated from aggregates was almost 2-fold higher in WAVE bioreactors (76.0 ± 5.8 %) compared to stirred tank cultures (43.9 ± 6.6, Fig. 4c, Table 1). Consequently, only 2 days of antibiotic treatment were sufficient to obtain a 97.6 % pure CM population in WAVE bioreactors (Fig. 4c, Table I). In contrast, at day 11 of differentiation, aggregates in stirred tank bioreactors were not yet completely pure and additional 5 days of antibiotic treatment were required to generate a 97.4 % pure CM population (Fig. 4a, c). In terms of CM productivities, in optimized stirred tank cultures approximately 0.430 L medium throughput (total volume of medium used during the process) resulted in the generation of 0.1x10^9^ CMs, which correspond to a coefficient of 0.2x10^9^ CMs/L. On the other hand, in WAVE bioreactor 2.3x10^9^ CMs were produced in 1.5 L medium throughput (1 L until day 9, 0.5 L medium exchange at day 9) obtaining 1.5x10^9^ CMs/L (Table 1). Overall, iPSC differentiation in WAVE bioreactors resulted in an improvement of about 40 % in CM yield (60.8 ± 0.7 CMs/input iPSC) and in a 5 times higher CM production per liter of culture medium throughput (CMs/L) in comparison to optimized stirred tank cultures (Fig. 4d, Table 1).

RT-PCR analysis showed that early mesoderm, cardiac progenitor and cardiac-specific genes were expressed earlier and to a higher extent in aggregates from WAVE cultures than in stirred cultures (Fig. 4e compare to Fig. 3e). The expression of the pluripotency gene Oct4 decreased more rapidly in aggregates cultured in WAVE bioreactors reaching very low levels on day 6 of differentiation and undetectable levels from day 8–9 onwards (Fig. 4e–f). In contrast, the Oct4 transcripts were still expressed at this stage of differentiation in stirred cultures and were not detectable only in purified CMs (Figs. 3e, 4f). Additionally, in WAVE cultures the peak in the early mesodermal gene expression (assayed by the expression of T-Brachyury) occurred earlier (day 4, Fig. 4e) than in stirred tank cultures (day 6, Fig. 3e). By day 6, the cardiac mesodermal (PDGFRα) and cardiac progenitor (cKit and Nkx2.5) genes were significantly higher expressed in WAVE than in stirred tank bioreactor cultures. The expression of cardiac specific markers was also detected earlier in WAVE cultures and increased gradually in later stages of differentiation (Fig. 4e). Importantly, the greater band intensities of cardiac transcripts Nkx2.5, cTnT and Myl7 observed in WAVE cultures before antibiotic selection (day 9) indicate that these cultures contained a substantially higher proportion of CMs than stirred bioreactor cultures (Figs. 3e, 4e). RT-qPCR analyses confirmed that by day 9 of differentiation the expression of these three cardiac markers was significantly higher (1.3, 1.6 and 1.6-fold, respectively) in WAVE cultures than in stirred tank bioreactors (Fig. 4f). At the end of antibiotic selection, the expression of cTnT and Myl7 were very similar in both bioprocesses (Fig. 4f) confirming the similar degree of CM purity in both groups (Table 1).

**Fig. 5:**
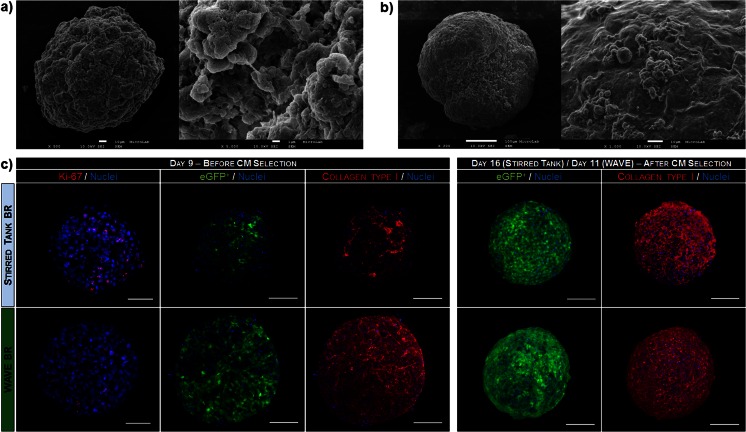
Scanning-electron and confocal microscopy of iPSC-derived cell aggregates and cardiospheres. **a**–**b**. SEM micrograph of differentiating cell aggregates collected from stirred tank (**a**) and WAVE (**b**) bioreactors at day 9 of differentiation. Higher magnification images evidence the differences in aggregate surface topography. **c**. Immunofluorescence and confocal microscopy images of the whole-mount day 9 aggregates (*left panel*) and cardiospheres collected at the end of selection process (*right panel*). The frequency and distribution of eGFP-positive (green), Ki-67 (*red*) and collagen type I-positive cells (*red*) is shown. Nuclei were labeled with DAPI (*blue*). *Scale bars*: 100 μm

Phase-contrast and scanning-electron microscopy (SEM) analysis of aggregates on day 9 of differentiation (i.e. before cell lineage selection) revealed that aggregates from WAVE and stirred tank bioreactor cultures differed in size and morphology (Fig. 5a–b, Supplementary Table I/Fig. III). The mean size of aggregates cultured in WAVE bioreactors was higher (440.04 ± 107.89 μm) and they were less spherical and more elongated than aggregates from stirred tank bioreactors (384.41 ± 124.80 μm) (Supplementary Table I, Supplementary Fig. III). This higher size and reduced “sphericity” observed in the aggregates cultured in the WAVE bioreactor might be justified by the different hydrodynamic environment promoted by this type of bioreactor. Additionally, aggregates from WAVE bioreactors showed a smooth outer surface (Fig. 5b), whereas aggregates from stirred tank cultures exhibited a looser texture and a rough surface in which cell-cell contacts could be discerned at a higher magnification (Fig. 5a). Previous studies demonstrated that during cardiac differentiation of ESCs, the extracellular matrix (ECM) mainly composed of collagen type I is deposited on the surface of differentiating aggregates providing for a smoother aggregate surface topography [27, 42]. Thus, our findings may suggest that prior to CM selection the aggregates cultured in WAVE bioreactors present a higher deposition of extracellular matrix (ECM) than stirred tank aggregates. To further confirm these observations, we stained the whole-mount day 9 aggregates with collagen type I antibody to determine the amount and distribution of this ECM component by confocal microscopy. This analysis clearly revealed that aggregates from WAVE bioreactors contain considerably higher levels of collagen type I than aggregates from stirred tank bioreactors (Fig. 5c, left panel) and that, based on the fraction of eGFP-positive cells in whole aggregates, the WAVE aggregates presented higher CM purity than the aggregates derived in stirred tank bioreactors, before induction of CM selection. Moreover, at this timepoint a lower percentage of proliferative cells (Ki-67 positive cells) was observed in aggregates derived from WAVE bioreactors when comparing with aggregates from stirred tank bioreactor cultures (approximately 20 % vs. 58 % of the cells, respectively, Fig. 5c). Taken together, our data show that iPSCs differentiate into CMs in WAVE bioreactors with faster kinetics and higher efficiency than in stirred tank bioreactors. At the end of the differentiation process and the puromycin selection procedure (day 16 for stirred tank and day 11 for WAVE bioreactor), collagen type I staining was similar in intensity and distribution in aggregates from both types of cultures (Fig. 5c, right panel).

### Structural Properties and Action Potential (AP) Parameters of CMs Generated in Stirred Tank and WAVE Optimized Bioprocesses

Before CM selection, some aggregates were dissociated into single cells and seeded in static culture plates for structural analyses. Immunofluorescence staining for sarcomeric α-actinin, as well as the distribution of α-MHC protein (eGFP staining), indicate that cells from WAVE cultures were more elongated and showed more organized sarcomeric structures, by day 9, when compared to cells from stirred tank cultures (Supplementary Fig. IV). These results are in agreement with the findings of other studies showing that cyclic tensions promote elongation of the cell membrane, orientation of actin filaments and a higher structural organization [33, 39].

**Fig. 6:**
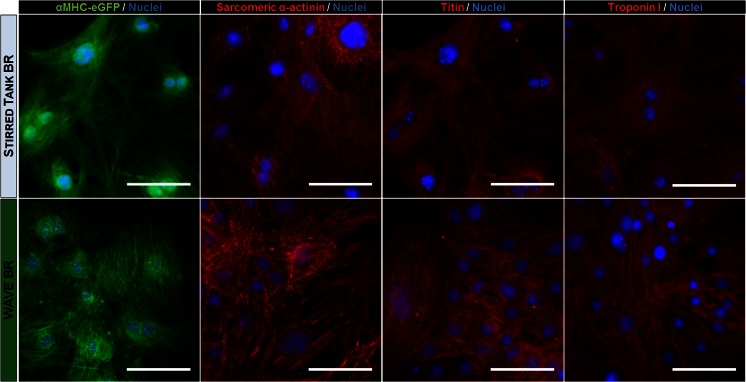
Structural properties of CMs generated in stirred tank and WAVE optimized bioprocesses. Cardiospheres were dissociated into single cells, plated on 2D plates and after being cultured for up to 2 weeks, stained for CM markers. Immunocytochemical analysis of eGFP-positive CMs (*green*) using sarcomeric α-actinin, titin and troponin I (*red*) antibodies. Nuclei are counterstained with DAPI (*blue*). *Scale bars*: 50 μm

After the differentiation and selection process, beating cardiospheres (Supplementary Movie II) were also dissociated into single cells and seeded in 2D plates for structural and functional characterization. Monolayers of pure CMs were obtained and cells maintained their spontaneous beating activity, which became synchronized over culture time (Supplementary Movie III). Immunocytochemical analysis of cardiac-specific proteins α-MHC, sarcomeric α-actinin, titin and troponin I revealed that iPSC derived-CMs produced in both bioreactor systems stained positive for these cardiac structural proteins (Fig. 6), presenting an organized striated pattern typical of CMs.

**Fig. 7:**
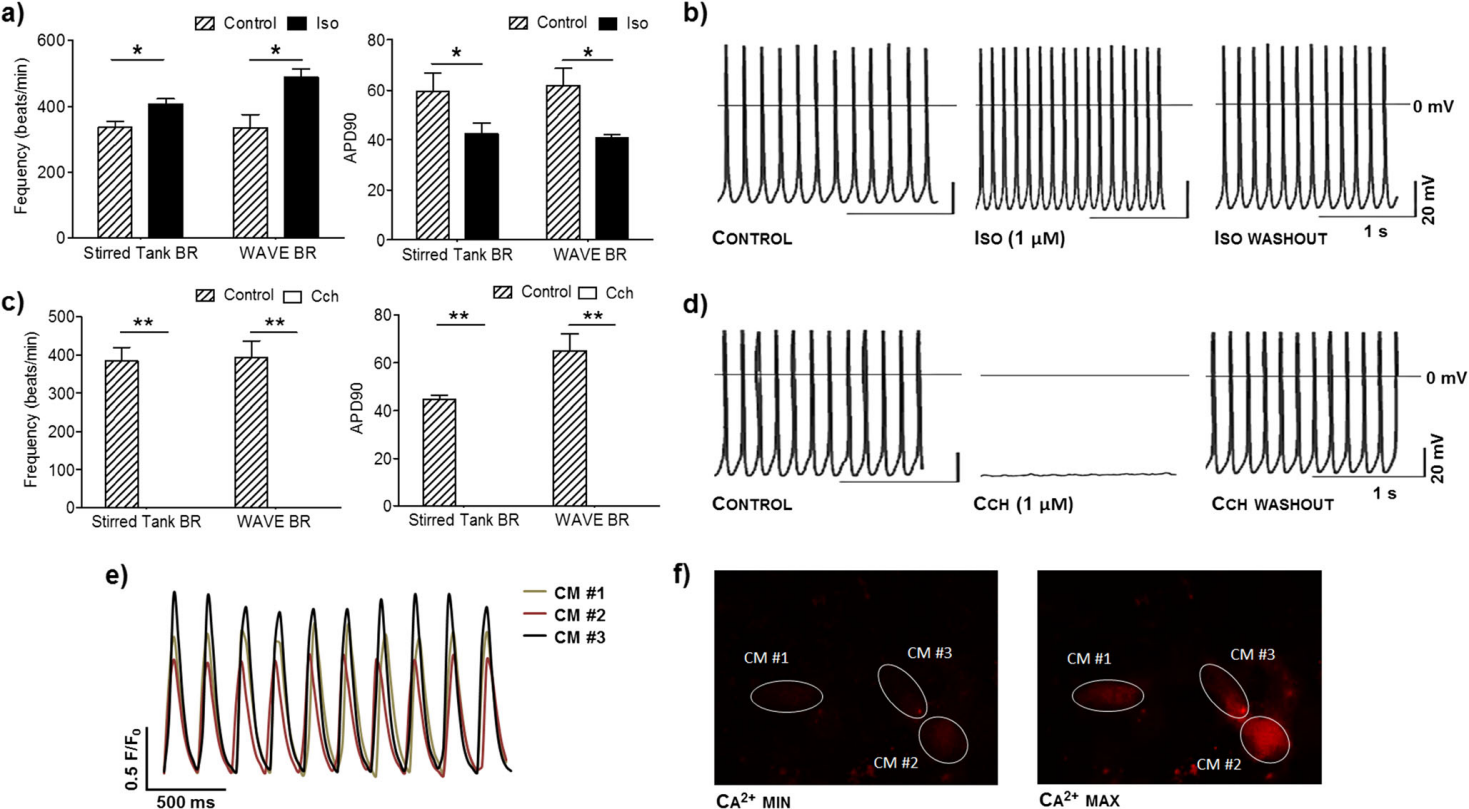
Functional characterization of CMs produced in stirred tank and WAVE bioreactors. **a**–**d**. Pharmacological response of CMs. Effect of 1 μM Isoproterenol (Iso, **a**–**b**) and 1 μM Carbachol (CCh, **c**–**d**) on beating frequency and on action potential (AP) duration at 90 % repolarization (APD90) in iPSC-derived CMs. **b**, **d**. Representative recordings of APs from WAVE bioreactor-derived CMs. APs were recorded before the application of the drug (Control) in the presence of the drug (Iso/CCh), and after washout of the drug (washout). **e**–**f**. Calcium transients of CMs produced in WAVE bioreactor. **e**. Graphical representation of calcium level cycling, during CMs contraction, determined by confocal imaging of the cell permeant calcium indicator dye Rhod-3, for three different cells. **f**. Pseudo-color images show minimal (Ca^2+^ min, left image) and maximal (Ca^2+^ max, right image) Rhod-3 fluorescence intensity in the three analyzed cells. Significantly different, *P* < 0.05 (*) and *P* < 0.01 (**)

In order to assess the electrophysiological properties of CMs generated in both bioreactor systems, AP recordings in single CMs were performed. The great majority of puromycin selected CMs derived from both bioprocesses displayed an atrial-like AP morphology (stirred tank: 100 %, WAVE: 92 %). These findings are also in agreement with the enhanced expression of atrial transcripts (Myl7) and reduced expression of the transcripts specific for the pacemaker (HCN4) and ventricular-like phenotypes (Myl2) detected in the final differentiated population by RT-PCR (Figs. 3e and 4e). The AP characteristics of purified CMs generated in WAVE and stirred cultures were very similar showing comparable maximum diastolic potential (MDP), beating frequency, AP upstroke velocity (V_max_), velocity of diastolic depolarization (V_dd_) and AP duration (APD) (Supplementary Table II). Additionally, the CMs generated in WAVE and stirred tank bioreactor cultures exhibited AP parameters similar to the ones described in literature for atrial-like late stage development fetal CMs (day 16–19 of differentiation), such as MDP (−63.3 ± 1.3 mV), frequency (234 ± 19 beats/min), V_max_ (27.9 ± 0.7 V/s) and V_dd_ (0.115 ± 0.017 V/s) [11]. Besides exhibiting largely indistinguishable AP properties, CMs produced in both bioprocesses also responded similarly to adrenergic and muscarinic agonists, isoproterenol and carbachol, respectively. When isoproterenol was administered to CMs a significant increase of AP frequency and a shortening of APD at 90 % of repolarization (APD90) was observed (Fig. 7a). Moreover, positive chronotropic effects of isoproterenol were reversible upon washout (Fig. 7b). In contrast, the administration of carbachol, a synthetic acetylcholine analog, evoked a significant reduction in the beating rate in CMs from stirred tank and WAVE bioreactors, which was also reversible upon washout (Fig. 7c–d).

In order to further evaluate the functional integrity of CMs produced in WAVE bioreactors we performed real-time intracellular calcium imaging with the calcium indicator dye Rhod-3. This analysis revealed that distinct iPSC-derived CMs from a beating monolayer present synchronized oscillatory patterns of intracellular calcium concentrations (Fig. 7e–f). Supplementary Movie IV shows a representative live imaging recording of whole-cell calcium transients for contracting CMs produced in WAVE bioreactors.

Overall, these results show that CMs produced using the protocol developed in this work exhibit intact molecular, structural and functional properties.

## Discussion

In this study we developed a new method for mass production of murine iPSC-derived CMs using environmentally controlled bioreactors. The expansion and CM differentiation of human and murine pluripotent stem cells in stirred tank bioreactors have been reported by our group [22] and others [25, 26, 43]. Aiming to further improve CM yields, we designed a bioreactor protocol that combine low oxygen concentrations with a cyclic mechanical rich environment by manipulating bioreactor hydrodynamics, more specifically the agitation type and profile. In this study, we showed for the first time that a DO of 4 % O_2_ tension, along with the use of either an intermittent stirring or a wave-induced agitation, favors iPSC differentiation towards the CM lineage. Using an intermittent agitation in stirred tank bioreactors, we were able to improve by 1000-fold CM yields (up to 44 CMs/input iPSC) when compared to normoxic continuously agitated cultures. Moreover, we demonstrated that the wave-induced agitation in combination with 4 % O_2_ tension from the beginning of iPSC differentiation increases the kinetics of cardiac differentiation, enabling a reduction of bioprocess duration by 5 days, and further improves the CM yields (60 CMs/input iPSC). To our knowledge, this is the first study reporting the use of the WAVE bioreactor for PSC culture. This type of bioreactor presents a simple design, appealing to either biological engineers or medical professionals and offers a low shear environment suitable for growth of sensitive cells such as stem cells [44].

Oxygen tensions and mechanical cues have been shown to mediate PSCs proliferation and differentiation. In our study, we showed that hypoxic exposure (4 % O_2_ tension) improves CM differentiation efficiencies by enhancing both cell proliferation and cardiomyogenesis when compared to normoxia conditions. This data is in accordance with previous studies [26], reporting that low DO concentrations enhanced the proliferation of ESCs and the expression of mesodermal, early and late cardiac-specific genes [26]. It is also established that CMs actively respond to mechanical cues from the environment in a frequency-dependent manner and that those cues can modulate electric remodeling, alterations in gene expression, autocrine and paracrine effects, and consequently cardiac tissue organization and development [39]. Cardiac cells experience mechanical strains with every heartbeat, i.e., at a pulsatile frequency close to 1 Hz [45]. Aiming to mimic the physiological environment, previous studies have applied mechanical stimulation at frequencies of 1 Hz on commercial or custom built devices to engineered cardiac tissues [31]. In our protocol, cells were subjected to distinct mechanical forces by manipulating the hydrodynamic environment in scalable readily available bioreactor systems. In stirred tank bioreactors, using an intermittent stirring, mechanical forces at frequencies close to 0.033 Hz were generated, whereas with a wave-induced agitation higher frequencies were reached (0.82–0.86 Hz). Thus, the differences in the temporal gene expression pattern and cardiac differentiation efficiency observed in stirred and WAVE cultures may be related to the frequency of mechanical loading applied in both systems. In accordance with literature, the effect of mechanical forces on cardiomyogenic differentiation is highly dependent on the experimental setup. Several variables including force magnitude, frequency, direction, duration of application, and at what stage of differentiation the force is applied, have been shown to affect cell fate decisions [39, 40, 46]. In fact, these aspects were noted to also be critical for the success of the protocol described herein. Our results suggest that an intermittent agitation profile with change of stirring direction is not suitable for iPSC differentiation into CMs (low CM numbers and purities were achieved at the end of the differentiation process). Also, higher cell lysis was observed during all culture time, suggesting that this agitation profile may have induced high shear rate for cells, compromising cell viability, proliferation capacity and differentiation efficiency. Herein we considered that the major mechanical stimulus induced to the cells is the hydrodynamic environment provided by an intermittent and wave-induced agitation. However, it should be noted that fluid shear stress per se might also influence cell behavior. The effect of shear stress on cell pluripotency and cardiomyogenesis has already been described. For example, mESCs exposed to laminar shear stress (4 days at 5 dyn/cm^2^) expressed higher levels of mesodermal markers [47]. On the other hand, fluid shear forces promoted by distinct types of impellers, such as single glass-ball stirring pendulum (1.52 dyn/cm^2^, 75 rpm) [48], pitched-blade impellers (2–5.2 dyn/cm^2^, 50–100 rpm) [49] and paddled impellers (4.5–7.8 dyn/cm^2^, 80–120 rpm) [50] have shown to maintain or increase the expression of pluripotency markers in PSC cultured in bioreactor systems. In WAVE bioreactors the mean values of shear stress (0.05 Pa, 0.5 dyn/cm^2^) in both 2 L and 20 L working volumes [51]) were considerably lower than the ones indicated above for stirred bioreactors. In accordance, we observed a 6-times lower fold increase in the cumulative LDH in WAVE bioreactor cultures, from day 2 to day 9, when compared to stirred tank bioreactor cultures (Supplementary Fig. II). These results reflect the reduced impact that fluid shear stress has on cell viability in WAVE bioreactor cultures. Future experiments using Computational Fluid Dynamics tools should be performed aiming at a deeper characterization of the hydrodynamic environment and quantification of the type and magnitude of the stresses generated in each bioreactor strategy.

It should be pointed out that mechanical forces, besides promoting elongation of the cell membrane and orientation of actin filaments [33, 39], regulate ECM synthesis, more specifically, result in enhanced synthesis of collagen, the most abundant protein in cardiac tissue [45]. In this study, we showed that by day 9 of differentiation, cultures that have experienced higher mechanical loading (WAVE cultures) are composed of cells with more organized sarcomeric structures, and aggregates with smoother surface topography due to a higher deposition of collagen type I.

Aside from different hydrodynamic environments, cells were cultured at 4 % O_2_ tension during different periods of time in these systems (in WAVE bioreactor from day 0 to day 11 and in stirred tank bioreactor from day 2 to day 16). Therefore, exposing cells to reduced oxygen concentrations from the beginning of differentiation could also have contributed to the beneficial effect on CM differentiation observed in WAVE cultures.

The establishment of pure CM preparations is imperative for future clinical application of these cells. Our finding that a hypoxic environment along with a wave-induced agitation enables the production of CMs at a purity of 76 % in nine days without growth factor- directed differentiation and without antibiotic selection represents a major advance towards development of robust and clinically applicable bioprocesses. To facilitate the optimization of the described method, we have used a transgenic murine iPSC line, in which the cardiac-restricted α-MHC promoter drives the expression of a puromycin resistance gene and eGFP. Therefore, antibiotic treatment, after day 9, resulted in the generation of an essentially pure CM population. In WAVE bioreactors, only two days were sufficient to achieve 97 % CM purity by puromycin selection whereas in stirred tank cultures 7 days of selection were needed to reach the same purity. CMs generated at faster kinetics may have higher therapeutic potential in their in vivo applications. Namely, Boheler and coworkers showed that ESC-derived CMs isolated at day 11 of differentiation, in contrast to cells obtained at day 16–18 of differentiation, were more resistant to hypoxia in vitro and survived longer following injection into healthy hearts of athymic nude mice [52]. These data suggest that early-stage CMs may have greater potential to engraft and improve the myocardial contractile function following infarction. Therefore, it is appealing to hypothesize that day 11 CMs obtained in WAVE bioreactors may exhibit higher survival and engraftment potential after intramyocardial transplantation than day 16 CMs generated in stirred tank bioreactors. In order to increase the safety of the final CM preparation, non-genetic cell-lineage purification protocols should be considered. Procedures involving density-gradient centrifugation [53], the use of a mitochondrial dye [54] or antibodies targeting cardiac-specific surface markers [55–57] have been established for CM enrichment and can be combined with the developed bioprocess. However, these methods are either labor intensive or expensive which limit their scalability. Non-genetic CM lineage purification strategies based on distinct metabolic requirements of CMs and non-CM cell types have been described. For example, it has been shown that CMs survive in serum-free medium [58, 59] or in glucose-depleted culture medium containing lactate [18], while other cell types do not. In addition, selective elimination of remaining contaminating pluripotent cells could be achieved by treatment of final CM preparations with small molecules that do not compromise CM viability and functionality but are toxic to PSCs [60]. From a large-scale production perspective this type of approaches are more appealing and could be easily incorporated in our bioreactor protocol aiming at establishing a clinically scalable and cost-effective integrated bioprocess for CM differentiation and purification.

Another major requirement for the biomanufacturing of stem cell derivatives is to ensure that the final product fulfills the desired quality requisites for biomedical applications including phenotype, potency and functionality [21]. Here, we showed that CMs obtained in both bioreactor systems presented typical cardiac morphology, electrophysiology, hormonal response to β-adrenergic and muscarinic receptor stimulation and rhythmic intracellular calcium transients. The most predominant CM subtype observed in both bioprocesses was the atrial-like phenotype, which was largely influenced by the type of cardiac specific promoter that was driving the expression of antibiotic resistance gene in our genetically engineered cells. The α-MHC promoter is active mostly in atrial regions during embryonic and early fetal development [61], therefore it seems that the selection strategy used in this work is favoring the production of immature fetal-like cells that present action potentials resembling those found in atrial cells and that have not yet assumed more recognizable cardiac chamber subtypes. Future efforts should be directed towards improving the electrophysiological maturity of the produced CMs and enhancing cardiac subtype specification, for example by extending the time in culture after the selection process.

In conclusion, this study describes a robust and scalable protocol for differentiation of murine iPSC into CMs in controlled hypoxic and mechanical environment. In this work we have focused on murine iPSC as a model system for bioprocess development, and on a simple non-directed differentiation protocol to clearly identify the impact of each culture condition tested on CM differentiation. Although human and mouse PSC lines utilize overlapping developmental pathways, much optimization is required when translating protocols between species. We are aware that further optimization to our protocol will be required when translating to human PSCs. For example the use of chemically defined and serum-free media supplemented with cytokines will be of major importance. Nevertheless, it is our conviction that the bioreactor protocol herein described (i.e. the controlled hypoxic and specific hydrodynamic environment promoted by an intermittent stirring or a wave induced agitation) will be able to further improve the protocols already reported for human PSC differentiation towards CMs by enhancing culture homogeneity, process reproducibility, CM yields and productivities. It is widely known that the use of chemically defined media and growth-factor supplements considerably increases the cost of the differentiation process. Thus, the development of cost-effective bioprocesses is also highly dependent on increasing the number of CMs generated per liter of culture medium throughput (CMs/L). Here, by controlling key environmental conditions in cardiac development, we were able to produce up to 1.6x10^9^ CMs/L in a single WAVE 11-days bioreactor run. To our knowledge this is the highest value that has been reported to date [43]. Hopefully, our achievements will also contribute for the development of affordable bioprocesses for mass production of human PSC-derived CMs, by reducing the need/amount of expensive cytokine inductive cocktails and/or the cardiac differentiation protocol duration.

## Electronic supplementary material

Below is the link to the electronic supplementary material.

Supplementary Movie I Differentiating aggregates from WAVE bioreactors. Movie was taken at day 9 of the differentiation process, with 200X magnification. This video demonstrates that by day 9 almost all aggregates present contractile areas. (MPG 1966 kb)

Supplementary Movie II Beating cardiosheperes obtained in WAVE bioreactors. Movies were taken at day 11 of the differentiation process, with 200X magnification. Brightfield video shows beating cell aggregates and the green fluorescence video shows pure eGFP-positive aggregates. (MPG 1592 kb)

Supplementary Movie III Contracting monolayers of iPSC-derived CMs produced in WAVE bioreactors. Movies were taken with 200X magnification. Brightfield video shows a synchronized beating through the cell monolayer and the green fluorescence video shows a pure eGFP-positive monolayer. (MPG 1318 kb)

Supplementary Movie IV Calcium flux across a contraction cycle. Movies were taken with 200X magnification. Red fluorescence represents the calcium transients. (AVI 12894 kb)

ESM 1 (DOCX 2184 kb)
