# Supplementary material for: Combining Hypoxia and Bioreactor Hydrodynamics Boosts Induced Pluripotent Stem Cell Differentiation Towards Cardiomyocytes
Source: Stem Cell Rev. 2014 Jul 15;10(6):786–801. doi: 10.1007/s12015-014-9533-0 (PMC4225049; doi:10.1007/s12015-014-9533-0)
Supplement: Supplementary file 5 — (DOCX 2184 kb) [file 12015_2014_9533_MOESM5_ESM.docx]

**SUPPLEMENTAL MATERIAL**

**Detailed Methods**

**Evaluation of cell concentration and viability**

***Cell membrane integrity assay***

The qualitative assessment of the cell membrane integrity during culture was done using the enzyme substrate fluorescein diacetate (FDA; Sigma-Aldrich, Germany) and the DNA intercalating dye propidium iodide (PI; Sigma-Aldrich, Germany) as previously described.[1]

***Trypan blue exclusion assay***

Cell aggregates were dissociated to single cells by a 5-7 minutes incubation with 0.25% (w/v) Trypsin-EDTA (Invitrogen, UK) at 37°C. Cell concentration and viability was assessed by the trypan blue exclusion method using a 0.1% (v/v) solution prepared in PBS and counting cells in a Fuchs-Rosenthal haemacytometer (Brand, Wertheim, Germany).

***Lactate dehydrogenase activity***

The extent of cell lysis during iPSC differentiation was assessed by measuring the activity of the intracellular enzyme lactate dehydrogenase (LDH) in the culture supernatant. LDH activity was determined by following spectrophotometrically (at 340 nm) the rate of oxidation of NADH to NAD^+^ coupled with the reduction of pyruvate to lactate. The specific rate of LDH release (qLDH, U.day^-1^.cell^-1^) was calculated for every time interval using the following equation: qLDH = ΔLDH/(Δt.ΔX_V_), where ΔLDH (U) is the change in LDH activity over the time period Δt (day) and ΔXv (cell) is the average of total cells during the same time period. The cumulative value (qLDH_cum_) was estimated by qLDH_cum t+1_ = qLDH_cum t_ + qLDH _t +1_.

**Evaluation of aggregate concentration**

The number of total aggregates and beating/eGFP-positive aggregates was determined in 4-6 wells of a 24-well plate containing 250-400 μL aliquots of bioreactor culture samples by using an inverted-microscope (DMI6000, Leica, Germany).

**Evaluation of aggregate size, elongation and roundness**

The aggregate size was determined using an inverted-microscope (DMI6000, Leica, Germany) by measuring two perpendicular diameters of each aggregate, from a minimum of 30 aggregates. These measures were used to calculate the average diameter of each aggregate. The elongation and roundness of aggregates was analyzed using measurement analysis tool of ImageJ software. Elongation (or aspect ratio) is defined by the ratio between the major and the minor axis. Roundness is the measure of how closely the shape of an object approaches that of a circle and is defined by the following: $4\times\frac{Area}{\pi\times{(Major axis)}^{2}}$. Both, elongation and roundness parameters were analyzed from a minimum of 30 aggregates per condition.

**Monitorization of CM differentiation**

***Flow cytometry***

After dissociation of aggregates or cardiospheres with 0.25% (w/v) Trypsin-EDTA as described above, cells were re-suspended in washing buffer (PBS with 5% (v/v) FBS) and the percentage of live (as assessed with PI dye), eGFP-positive cells were analyzed in a CyFlow® space (Partec GmbH, Germany) instrument, registering 10000 events/sample. The results throughout this analysis constituted a measurement of the percentage of CMs in culture at each time point, reflecting the purity level of the culture.

***Semiquantitative and quantitative RT-PCR***

Aggregates or cardiospheres were washed with PBS and centrifuged at 300x*g* for 5 min. The pellet containing 10^6^ cells was snap-frozen by immersion in liquid nitrogen. Total RNA was extracted using the High Pure RNA Isolation Kit (Roche, Germany) and reverse transcription was performed with High Fidelity cDNA Synthesis Kit (Roche, Germany) using 200ng of RNA, following manufacturer’s instructions.

Gene expression profiles of various markers during cardiac differentiation were analyzed by semiquantitative reverse transcriptase polymerase chain reaction (RT-PCR) using the DreamTaq Green PCR Master Mix (Thermo Scientific, USA). Analyses were performed in 20 µl reactions containing 0.5 µM of each primer and 1 µl of undiluted cDNA template. PCR products (10 µl) were electrophoretically separated on 1.5 % agarose (Invitrogen, UK) gel and ethidium bromide-stained bands were detected with a CCD camera using Intas UV-System and Intas GDS application (Intas, Germany). The primer sequences used in RT-PCR are listed in Online Table III.

Quantitative RT-PCR was performed in triplicate, for each sample and each gene, using SYBR Advantage qPCR Premix (Clontech, USA) in 10 μl reactions with 1:25 diluted cDNA template and 0.2 μM of each primer. The reactions were performed in 384-well plates using AB 7900HT Fast Real Time PCR System (Applied Biosystems, Germany). Cycle threshold (Ct’s) and melting curves were determined by SDS 2.1 Software. All data was analyzed using the 2^-ΔΔCt^ method for relative gene expression analysis. Changes in gene expression were normalized to GAPDH gene expression as internal control.

**Evaluation of cell growth and differentiation towards CMs**

**Cell expansion fold**

The cell expansion fold was evaluated based on the ratio X_d9_/X_d0_, where X_d9_ is the total cell number at day 9 (before purification) and X_d0_, the total initial cell number. In Stirred tank bioreactors X_d0_ represents the initial cell number corresponding to the 30000 aggregates inoculated *per* bioreactor at day 2.

**Final CM yield**

The final number of CMs was determined by flow cytometry of eGFP-positive cells at the end of the differentiation process. The CM yield was calculated by the number of eGFP-positive cells divided by the total initial number of iPSCs.

**CMs produced per liter of culture medium throughput**

The number of CMs produced per liter of culture medium throughput was determined by the number of eGFP-positive cells at the end of the process divided by the total volume of culture medium used throughout the differentiation process, considering all medium exchanges.

**Characterization of iPSC-derived CMs**

***Immunocytochemistry***

At the end of the differentiation process, aggregates were dissociated and plated on CellStart-coated 6-well plates. Adherent cells were fixed with 4% (w/v) buffered paraformaldehyde (pH 7.5) for 15 minutes at 37°C and permeabilized with 0.5 M ammonium chloride (Sigma-Aldrich), 0.25% Triton X-100 in PBS for 10 min at room temperature (RT). After blocking with 5% (v/v) FBS in PBS for 1 h at room temperature, cells were incubated with primary antibody overnight at 4°C in 0.8% (w/v) bovine serum albumin (BSA) in PBS. The primary antibodies used were anti-sarcomeric-α-actinin (1:800; clone EA-53, Sigma-Aldrich, Germany), anti-titin (E-2; 1:100 dilution; Santa Cruz Biotechnology, USA), anti-troponin I (1:100 dilution; Merck Millipore, Germany), and anti-atrial natriuretic peptide (ANP; 1:200 dilution; Merck Millipore, Germany). After two washing steps with PBS, samples were incubated for 60 min at RT with the secondary antibody anti-mouse-IgG1 AlexaFluor 594 (1:200 dilution in 0.8% (w/v) BSA in PBS; Invitrogen, UK). Nuclei were stained using Hoechst 33432 (1:5000 dilution in PBS; Sigma-Aldrich, Germany). Images were acquired using an inverted fluorescence microscope (DMI6000, Leica, Germany). For immunostaining of whole mounted aggregates, the permeabilization step was performed with 0.2% (w/v) fish skin gelatine and 0.1% (w/v) TX-100 in PBS for 2 h at RT. In this case, the anti-collagen type I (1:200 dilution, Abcam, UK) and the anti-Ki-67 (1:200 dilution, Merck Millipore, Germany) antibodies were used. Aggregates were visualized using spinning disk confocal microscope (Andor Revolution XD, Nikon Eclipse Ti-E, confocal scanner: Yokogawa CSU-x1).

***Scanning electron microscopy***

For scanning electron microscopy analysis, aggregates were washed twice in Sorensen’s Buffer (0.1M NaH_2_PO_4_, 0.1M Na_2_HPO_4_, pH 7.4), fixed with 4% (w/v) formaldehyde and 2.5% (w/v) glutaraldehyde solution for 1 hour at RT, followed by overnight incubation at 4°C. Samples were then washed with Sorensen’s Buffer again. After dehydration all samples were dried, coated in a sputter-coater with a layer of gold and observed under a scanning electron microscope (FEG-SEM: JEOL 7001F/Oxford INCA Energy 250/HKL).

***Detection of Ca^2+^ Transients***

Ca^2+^ imaging was performed according to the standard protocol provided in Rhod-3 - Calcium Imaging kit (Invitrogen). Briefly, cardiospheres were plated onto eight-well culture slides coated with CellStart. After one week in culture to promote cell adherence to the plate, the beating CMs were loaded with the cell permeant calcium indicator dye Rhod-3 for 1h at 37°C, washed twice in PBS, and incubated for 1h with a water-soluble reagent to reduce baseline signal and washed again in PBS. Cells were imaged live using a spinning disk confocal microscope (Andor Revolution XD). Fluorescence was measured by manually defining each region of interest and quantified in relation to baseline fluorescence (F/F0) using Micro-Manager 1.4 and ImageJ softwares.

***Electrophysiological characterization***

Purified cardiac clusters were washed twice with PBS and dissociated using 0.05% Trypsin/EDTA 2 days before measurement. Single CMs were plated on glass cover slips coated with 0.1% gelatin. The patch clamp-experiments were performed as described previously [2]. Briefly, the cover slips were placed into a recording chamber (37°C) and cells were continuously perfused with extracellular solution. Cell membrane capacitance was determined online using Pulse software (Heka Elektronik, Germany). Action potential (AP) recordings of spontaneously beating CMs were performed utilizing the whole-cell current-clamp technique with an EPC-9 amplifier (HEKA Elektronik) and operated through the Pulse acquisition software. Response to hormonal regulation was analyzed by administering 1 µM isoproterenol (Iso, Sigma-Aldrich, USA) or 1 µM carbachol (Cch, Sigma-Aldrich, USA).

**Statistical analysis**

For each culture system evaluated, at least three independent experiments were performed. The results were presented as the mean±standard deviation. For the electrophysiological characterization data are shown as mean±SEM. Statistical significance was assessed by one-way analysis of variance (ANOVA). Values of p<0.05 were considered statistically significant.

**Supplemental Figures**


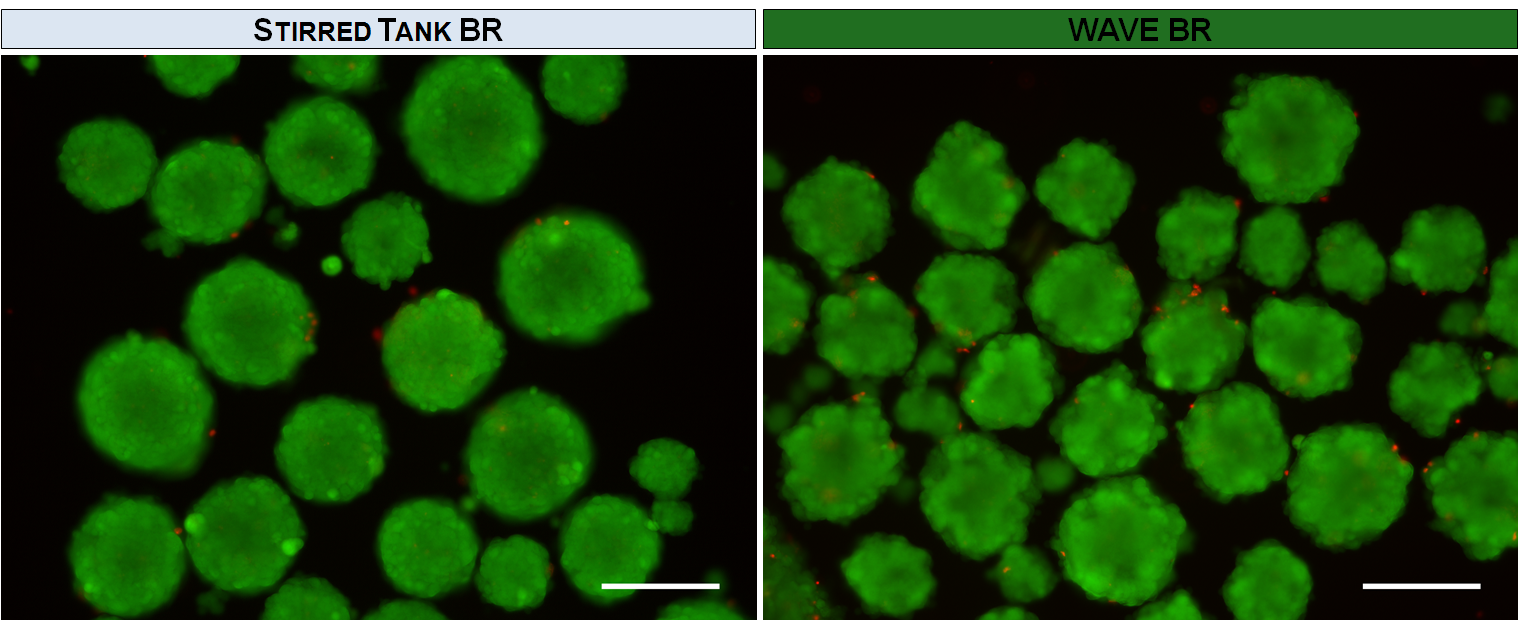


**Supplementary Fig. I. Viability analysis of cell aggregates from day 2 cultures**. Aggregates from both stirred tank and WAVE bioreactors were stained with fluorescein diacetate to detect live cells (green) and propidium iodide to identify dead cells (red). Scale bars: 200 μm.

**
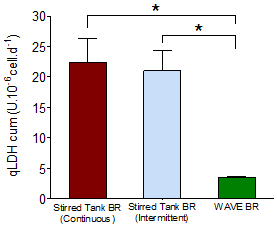
**

**Supplementary Fig. II. Cumulative LDH release in stirred tank and WAVE bioreactors.** Fold increase in the cumulative LDH release, from day 2 to day 9, in both stirred tank (operated under continuous and intermittent agitation without direction change) and WAVE bioreactors. Significantly different, P<0.05 (*).

**
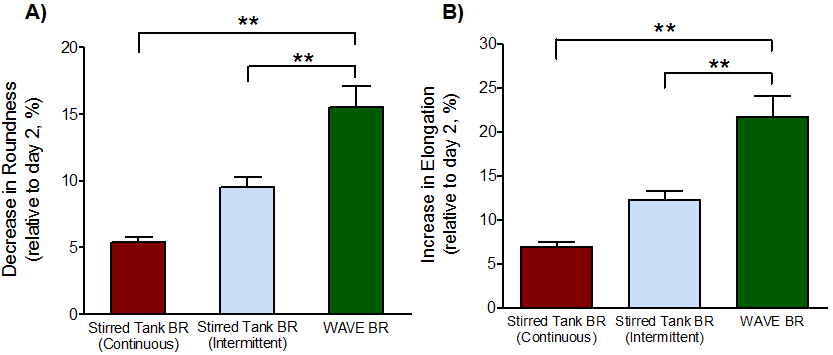
**

**Supplementary Fig. III. Elongation and roundness of aggregates from day 9 cultures.** Aggregates from day 9 cultures, in both stirred tank (operated under continuous and intermittent agitation without direction change) and WAVE bioreactors, were analyzed based on their elongation **(A)** and roundness **(B)**. Values were normalized to day 2 cultures. Significantly different, P<0.05 (*) and P<0.01 (**).

**
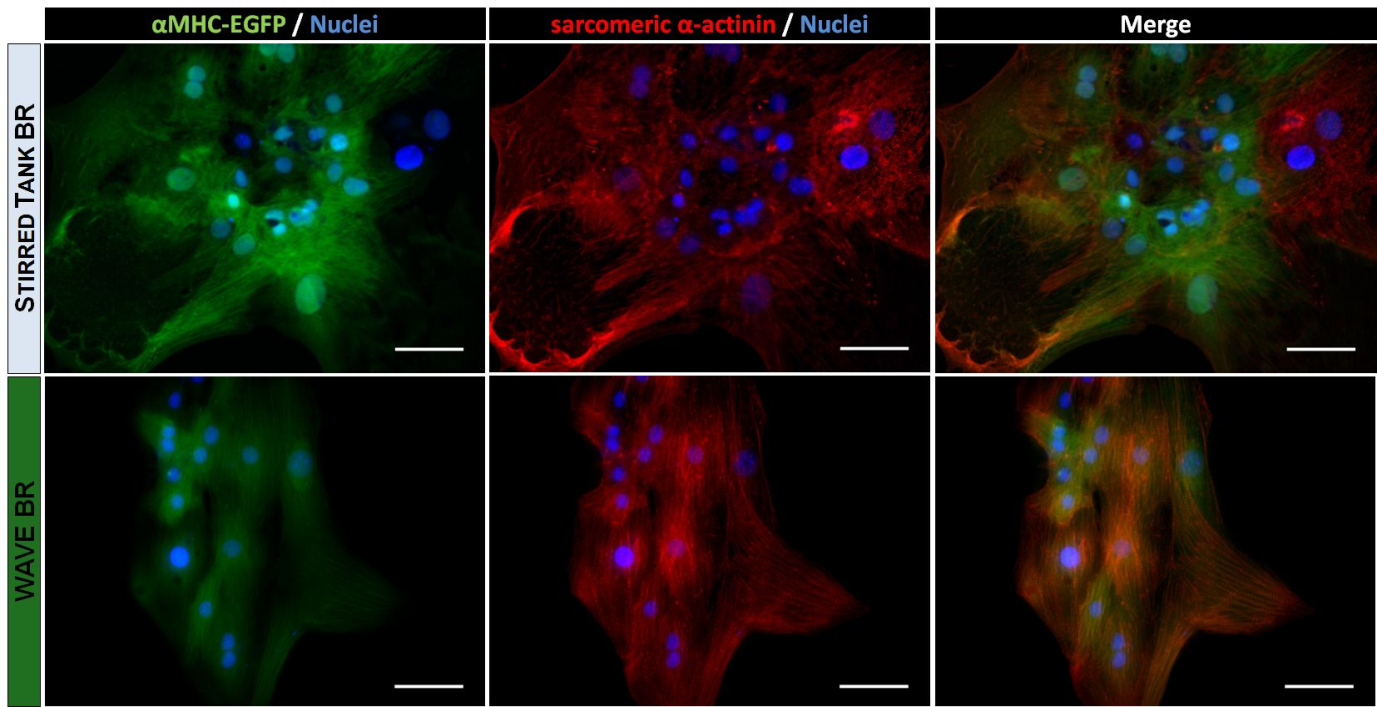
**

**Supplementary Fig. IV. Structural analysis of cells dissociated from day 9 aggregates cultured in Stirred tank and WAVE bioreactors.** Day 9 aggregates were dissociated into single cells, plated on 2D plates and stained with the sarcomeric α-actinin (red) antibody. Fluorescence images of αMHC-eGFP-positive cells (green) are also shown. Nuclei are counterstained with DAPI (blue). Scale bars: 50 μm.

**Supplemental Tables**

**Supplementary Table I. Mean aggregate size throughout time of iPSC-derived CMs produced in both automated stirred tank and WAVE bioreactor systems.**

| Time (day) | **Stirred Tank BR (Continuous)** | **Stirred Tank BR (Intermittent)** | **WAVE BR** |
| --- | --- | --- | --- |
| 2 | 142.65±69.85 | 155.34±55.57 | 169.37±45.90 |
| 4 | 147.25±68.90 | 181.04±66.94 | 301.79±63.08 |
| 7 | 307.38±116.39 | 277.84±131.39 | 371.23±74.79 |
| 9 | 357.64±113.30 | 384.41±124.80 | 440.04±107.89 |

**Supplementary Table II. Action potential properties of iPSC-derived CMs produced in both the automated stirred tank and WAVE bioreactor systems.**

| **Culture system** | ***N*** | **MDP (mV)** | **Frequency (beats/min)** | **V_max_ (dE/dt) (V/s)** | **V_dd_**  **(V/s)** | **APD90**  **(ms)** | **APD50**  **(ms)** | **APD90/APD50** |
| --- | --- | --- | --- | --- | --- | --- | --- | --- |
| **Stirred tank bioreactor*** | 22 | -59.8±1.0 | 321.9±23.3 | 26.5±1.8 | 0.1±0.0 | 84.4±16.4 | 18.1±1.1 | 5.3±1.3 |
| **WAVE bioreactor*** | 25 | -58.0±1.2 | 296.4±25.1 | 21.0±2.4 | 0.1±0.0 | 84.0±7.3 | 23.0±1.9 | 4.1±0.5 |

* Data are presented as means ± SEM.

*Abbreviations:* N, total number of cells analyzed; MDP, average maximum diastolic potential for action potentials during the time period examined; V_max_, the maximum rate of rise of the action potential upstroke; V_dd_, velocity of diastolic depolarization; APD90, action potential duration at 90% of repolarization; APD50, action potential duration at 50% of repolarization.

**Supplementary Table III. Primers for RT-PCR and quantitative RT-PCR**

| **Genes** | **Sequences (5’-3’)** | **Size (bp)** | **NCBI ID** |
| --- | --- | --- | --- |
| *GAPDH* | **F:** ACCTTGCCCACAGCCTTG | 142 | NM_001289726.1   NM_008084.3 |
|  | **R:** GGCTCATGACCACAGTCCAT |  |  |
| *Tnnt2* | **F:** GGTGCCACCCAAGATCCCCG | 199 | NM_001130174.1 |
|  | **R:** AATACGCTGCTGCTCGGCCC |  |  |
| *Nkx2.5* | **F:** CAGCCAAAGACCCTCGGGCG | 142 | NM_008700.2 |
|  | **R:** TGCGCCTGCGAGAAGAGCAC |  |  |
| *HCN4* | **F:** TGCTGTGCATTGGGTATGGA | 337 | NM_001081192.1 |
|  | **R:** TTTCGGCAGTTAAAGTTGATG |  |  |
| *MLC-2v* | **F:** TGCCAAGAAGCGGATAGA | 328 | NM_010861.3 |
|  | **R:** CAGTGACCCTTTGCCCTC |  |  |
| *mLC-2a* | **F:** AGTAGGAAGGCTGGGACCCG | 306 | NM_022879.2 |
|  | **R:** CTCGGGGTCCGTCCCATTGA |  |  |
| *T-Brachyury* | **F:** ctgcgcttcaaggagctaac | 91 | NM_009309.2 |
|  | **R:** CCAGGCCTGACACATTTACC |  |  |
| *KIT* | **F:** ATTATGAACGCCAGGAGACG | 497 | NM_001122733.1 NM_021099.3 |
|  | **R:** GAATCCCTCTGCCACACACT |  |  |
| *PDGFRA* | **F:** CGTCAAAGGGAGGACGTTCA | 370 | NM_011058.2,  NM_001083316.1 |
|  | **R:** GACGAAGCCTTTCTCGTGGA |  |  |
| *AFP* | **F:** CCCACTTCCAGCACTGCCTGC | 374 | NM_007423.4 |
|  | **R:** GGCTGCAGCAGCCTGAGAGT |  |  |
| *OCT4* | **F:** CATGTGTAAGCTGCGGCCC | 268 | NM_001252452.1 |
|  | **R:** GCCCTTCTGGCGCCGGTTAC |  |  |

**Supplementary References**

1. Serra, M., Correia, C., Malpique, R., et al. (2011). Microencapsulation Technology: A Powerful Tool for Integrating Expansion and Cryopreservation of Human Embryonic Stem Cells. *PLoS ONE*, *6*(8), 1–13.

2. Kuzmenkin, A., Liang, H., Xu, G., et al. (2009). Functional characterization of cardiomyocytes derived from murine induced pluripotent stem cells in vitro. *FASEB journal*, *23*(12), 4168–4180.
